# Effects of hydroalcoholic extract of Coriandrum sativum on oxidative damage in pentylenetetrazole-induced seizures in rats

**Published:** 2015-04-04

**Authors:** Reza Karami, Mahmoud Hosseini, Toktam Mohammadpour, Ahmad Ghorbani, Hamid Reza Sadeghnia, Hassan Rakhshandeh, Farzaneh Vafaee, Mahdi Esmaeilizadeh

**Affiliations:** 1 Student Research Committee, School of Medicine, Mashhad University of Medical Sciences, Mashhad, Iran; 2 Neurocognitive Research Center AND Department of Physiology, School of Medicine, Mashhad University of Medical Sciences, Mashhad, Iran; 3 Neurogenic Inflammation Research Center AND Department of Physiology, School of Medicine, Mashhad University of Medical Sciences, Mashhad, Iran; 4 Pharmacological Research Center of Medicinal Plants AND Department of Pharmacology, School of Medicine, Mashhad University of Medical Sciences, Mashhad, Iran; 5 Esfarayen Faculty of Medical Sciences, Esfarayen, Iran

**Keywords:** Coriandrum sativum, Pentylenetetrazole, Seizures, Rat, Oxidative Stress, Brain

## Abstract

**Background: **An important role for oxidative stress, as a consequence of epileptic seizures, has been suggested**.** Coriandrum sativum has been shown that have antioxidant effects**. **Central nervous system depressant effects of C. sativum have also been reported. In this study, the effects of hydroalcoholic extract of aerial parts of the plants on brain tissues oxidative damages following seizures induced by pentylenetetrazole (PTZ) was investigated in rats.

**Methods:** The rats were divided into five groups and treated: (1) Control (saline), (2) PTZ (90 mg/kg, i.p.), (3-5) three doses (100, 500 and 1000 mg/kg of C. sativum extract (CSE) before PTZ. Latencies to the first minimal clonic seizures (MCS) and the first generalized tonic-clonic seizures (GTCS) were recorded. The cortical and hippocampal tissues were then removed for biochemical measurements.

**Results: **The extract significantly increased the MCS and GTCS latencies (P < 0.01, P < 0.001) following PTZ-induced seizures**.** The malondialdehyde (MDA) levels in both cortical and hippocampal tissues of PTZ group were significantly higher than those of the control animals (P < 0.001). Pretreatment with the extract prevented elevation of the MDA levels (P < 0.010–P < 0.001). Following PTZ administration, a significant reduction in total thiol groups was observed in both cortical and hippocampal tissues (P < 0.050). Pre-treatment with the 500 mg/kg of the extract caused a significant prevention of decreased in total thiol concentration in the cortical tissues (P < 0.010).

**Conclusion: **The present study showed that the hydroalcoholic extract of the aerial parts of C. sativum possess significant antioxidant and anticonvulsant activities.

## Introduction

Epilepsy is a common neurological disease, which affects approximately 1% of the population.^1^ It is characterized by abnormal episodic bursts of electrical activity in neurons, which is followed by a significant impact on the behavior of the affected patients.^2^ An important role for oxidative stress both as a consequence and as a cause of epileptic seizures has been suggested.^3^ It has been reported that production of free radicals increases during seizures, which is lead to oxidative damage to lipids, DNA and susceptible proteins.^4^

Due to high levels of membrane lipid constituents, the central nervous system (CNS) is very susceptible to oxidative injury.^5^ In addition, oxidative damage plays a significant role in the pathogenesis of various CNS disorders and neurobehavioral impairments.^5^ The functional impairments of CNS, which occur during seizures have also been suggested to be at least in part, related to the brain tissues oxidative damages.^6^ Furthermore, the anticonvulsant activities of several agents with antioxidant effects such as melatonin, vineatrol, trans-resveratrol and alpha lipoic acid have been documented.^7^^,^^8^ There are also some reports that reactive oxygen species (ROS) may underlie the convulsant and neurotoxic effects of pentylenetetrazole (PTZ).^9^ The results of human and animal studies imply that epilepsy and seizures are lead to the brain tissues oxidative damages, especially in the cortical and hippocampal regions, which are accompanied with cognitive, learning and memory deficits.^4^^,^^6^^,^^8^^,^^10^

Medicinal plants are good sources to find new therapeutic agents for human diseases. Coriandrum sativum, an annual herb belonging to the Apiaceae family, has been reported to have a wide range of biological activities including sedative-hypnotic, antidiabetic, hypolipidemic, and hepatoprotective effects.^11^^-^^15^ Experimental studies have also revealed a strong antioxidant activity for C. sativum that is superior to the well-known antioxidant agents like ascorbic acid.^15^^-^^20^ In our previous work, we found that the hydroalcoholic extract of aerial parts of this plant bearing some compounds with the hypnotic effects.^21^ Regarding the antioxidant and CNS depressant effects of C. sativum, we aimed to evaluate the possible protective effects of aerial parts of the plant on PTZ-seizures and the brain tissues oxidative damages in rats.

## Materials and Methods

PTZ was purchased from Sigma-Aldrich Company (St. Louis, USA). Other chemical compounds such as thiobarbituric acid (TBA), trichloroacetic acid (TCA), hydrochloric acid (HCL), ethylenediaminetetraacetic acid (EDTA) and 2, 2'-dinitro-5, 5'-dithiodibenzoic acid (DTNB) were bought from Merck Company.

In this study, 40 virgin male Wistar rats, 250 ± 20 g in weight were used. The animals were maintained at the animal house under controlled conditions including 12 h light and dark cycle, 22-24 °C temperature and appropriate humidity with laboratory chow and water provided ad libitum.

The animals were randomly divided into five groups and treated (n = 8 in each group) as follows: (1) Control (saline), (2) PTZ, (3) C. sativum extract (CSE) 100 mg/kg (CSE 100) + PTZ, (4) CSE 500 mg/kg (CSE 500) + PTZ and (5) CSE 1000 mg/kg (CSE 1000) + PTZ. The doses were chosen regarding our previous study.^21^The number of animals was also based on our previous studies.^9^^,^^21^^-^^24^

The animals in groups 2-5 were treated intraperitoneally (i.p.) with saline or the extract 30 min before i.p. injection of a single dose (90 mg/kg) of PTZ. In our previous works, we showed that PTZ in this dose induces generalized tonic-clonic seizures (GTCS) in rats.^9^^,^^22^^,^^24^^,^^25^ The time interval between injection of the extract and PTZ was chosen regarding our previous work in which injection of the extract 30 min before injection of pentobarbital increased the sleeping time.^21^

The cortical and hippocampal regions were then removed for biochemical measurements. In the control group, saline was injected instead of both PTZ and CSE and the brain tissues were removed without inducing the seizures. All efforts were made to maintain the animals in good general health, in accordance with the European Communities Council Directive (2010/63/UE). All behavioral tests were conducted between 10:00 and 14:00 O'clock. Animal handling and all related procedures were confirmed by the Mashhad University of Medical Sciences, Iran, Ethical Committee.

The aerial parts (leaves, stems, twigs) of C. sativum were collected from Neyshabur, Iran. The identity of the plant was confirmed and for future reference a voucher specimen (10068) was deposited at the herbarium of School of Pharmacy (Mashhad University of Medical Sciences). To prepare the hydroalcoholic extract, the plant materials (50 g) were dried and extracted with 300 ml ethanol-water (70/30, v/v) using a Soxhalet apparatus. The extract reduced to dryness with a rotary vacuum evaporator (Stuart RE300, UK).^26^

In order to observe ictal behavior, PTZ was injected and the animals were placed in a Plexiglas arena (30 cm × 30 cm × 30 cm) on the day of the experiment. The animals were observed during 60 min after PTZ (90 mg/ kg) administration.^9^^,^^22^^,^^24^^,^^25^^,^^27^ The behavioral responses of the animals to PTZ administration were evaluated using these criteria: latency to the first minimal clonic seizure (MCS), incidence of MCS, latency to the first GTCS, incidence of GTCS, protection percentage against GTCS and protection percentage against mortality.^23^^-^^25^

After behavioral study, the rats were quickly decapitated under deep sodium pentobarbital anesthesia, their brains were removed and the cortical and hippocampal regions were separated and conserved for biochemical measurements. The animals were killed by a competent person with a minimum pain, suffering, and distress. The method was performed as set out in that Annex IV of the guidelines from Directive EU/2010/63 of the European Parliament.

For total thiol (SH) content measurement, the cortical and hippocampal regions were dissected on an ice-cold surface and homogenized in iced-cold phosphate-buffered saline to give 10% homogeny. Total SH groups were measured using DTNB as the reagent. This reagent reacts with the thiol groups to produce a yellow colored complex, which has a peak absorbance at 412 nm. Briefly, 1 ml Tris-EDTA buffer (pH = 8.6) was added to 50 μl of the brain homogenates, and the sample absorbance was read at 412 nm against Tris-EDTA buffer alone (A_1_). Then, 20 μl DTNB reagents (10 mm in methanol) were added to the mixture and after 15 min (stored in laboratory temperature), the sample absorbance was read again (A_2_). The absorbance of DTNB reagent was also read as a blank (B). Total thiol concentration (mm) was calculated from the following equation.^9^^,^^28^^-^^30^

Total thiol concentration (mM) = (A_2_-A_1_-B) × 1.07/0.05 × 13.6

Malondialdehyde (MDA) levels, as an index of lipid peroxidation, were also measured. MDA reacts with TBA as a TBA reactive substance to produce a red colored complex, which has a peak absorbance at 535 nm. The TBA/TCA/HCL reagent was added to the homogenates, and the solution was heated in a water bath for 40 min. After cooling, the whole solutions were centrifuged within 1000 g for 10 min. The absorbance was measured at 535 nm.^9^^,^^28^^-^^30^ The MDA concentration was calculated as follows: C (M) = Absorbance/(1.56 × 10^5^)

All data were expressed as mean ± standard error of the mean and analyzed by using ANOVA, followed by Tukey’s post-hoc comparison test. P < 0.0500 were considered to be statistically significant.

## Results

***Effect of C. sativum on PTZ-induced seizures***

**Figure 1:**
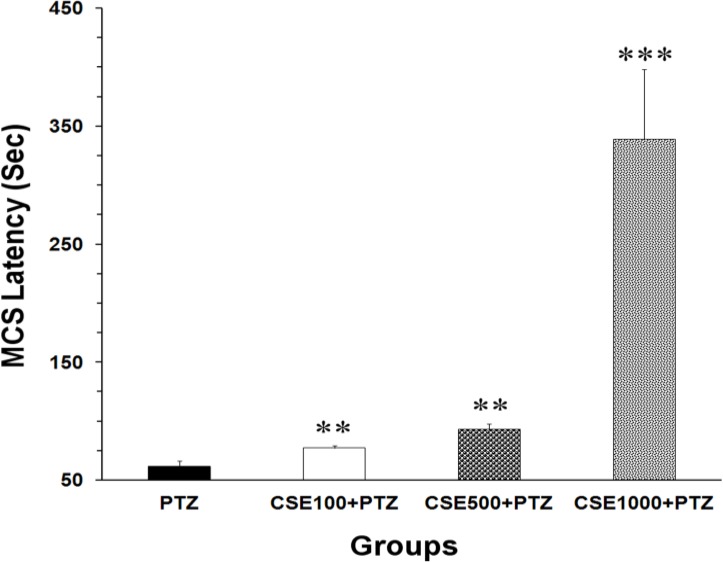
Latencies to minimal clonic seizures (MCS) onsets in pentylenetetrazole (PTZ), C. sativum extract (CSE) 100 mg/kg (CSE 100)-PTZ, CSE 500 mg/kg (CSE 500)-PTZ, CSE 1000 mg/kg (CSE 1000)-PTZ groups. The animals were treated with saline or CSE (100, 500 or 1000 mg/kg) before a single injection (90 mg/kg) of PTZ; ^**^ P < 0.010; ^***^ P < 0.001 as compared to PTZ group

All the animals in different groups (except the control group, which did not receive PTZ) showed MCS and GTCS following administration of a high dose of PTZ. Data analysis using one-way ANOVA showed that there was a significant difference between the groups in MCS latencies (F_3,28_ = 19.65, P < 0.0001). MCS latencies in the extract pre-treated groups were significantly higher than that of PTZ group. When compared with PTZ group (61.66 ± 4.76 s), 100, 500 and 1000 mg/kg of the extract significantly (P < 0.0100 to P < 0.0010) increased the MCS latencies to 77.3 ± 2.07, 93.5 ± 4.35 and 339.3 ± 58.96 s, respectively (Figure 1).

**Figure 2:**
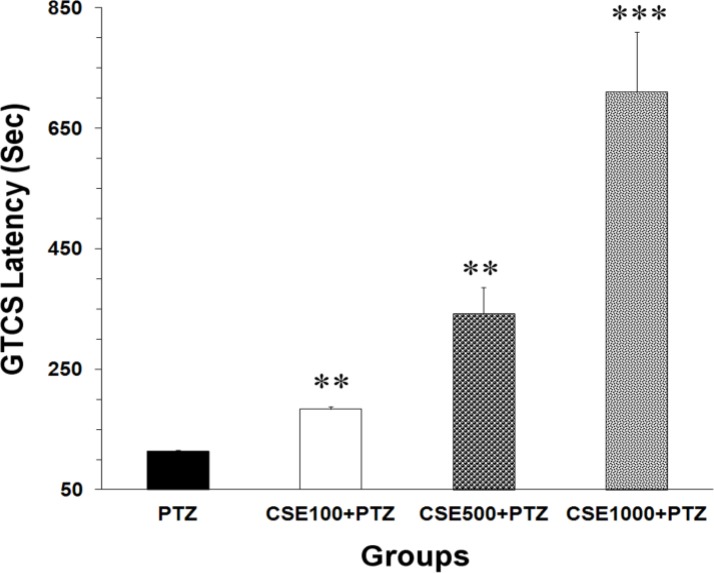
Latencies to generalized tonic-clonic seizures (GTCS) onsets in pentylenetetrazole (PTZ), C. sativum extract (CSE) 100 mg/kg (CSE 100)-PTZ, CSE 500 mg/kg (CSE 500)-PTZ, CSE 1000 mg/kg (CSE 1000)-PTZ groups. The animals were treated with saline or CSE (100, 500 or 1000 mg/kg) before a single injection (90 mg/kg) of PTZ; ^**^ P < 0.010; ^***^ P < 0.001 as compared to PTZ group

Data analysis using one-way ANOVA also showed that there was a significant difference between the groups in GTCS latencies (F_3,28_ = 24.13, P < 0.0001). The GTCS latencies in the animals, which had received 100, 500 and 1000 mg/kg of CSE before PTZ, were 183 ± 4.69, 341.88 ± 44.16, and 710.3 ± 98.84 c, respectively. All 3 doses of the extract significantly increased the GTCS latencies (P < 0.0100 to P < 0.0010) compared with PTZ group (114 ± 1.8 c) (Figure 2). There were no significant differences in mortality rate following PTZ administration between groups.

***Effect of C. sativum on*** ***brain tissues oxidative damage***

**Figure 3:**
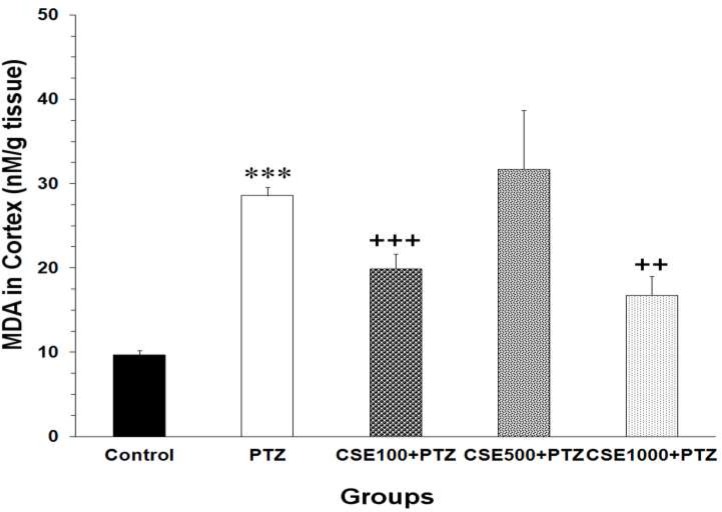
Comparison of the malondialdehyde (MDA) levels in cortical tissues of control, pentylenetetrazole (PTZ), C. sativum extract (CSE) 100 mg/kg (CSE 100)-PTZ, CSE 500 mg/kg (CSE 500)-PTZ, CSE 1000 mg/kg (CSE 1000)-PTZ groups. The animals were treated with saline or CSE (100, 500 or 1000 mg/kg) before a single injection (90 mg/kg) of PTZ; The animals in control group received saline instead of PTZ; ^***^ P < 0.001 as compared to control group; ^++^ P < 0.010; ^+++^ P < 0.001 as compared to PTZ group

Data analysis using one-way ANOVA showed that there was a significant difference between the groups in MDA concentrations of cortical tissues (F_4,35_ = 6.93, P < 0.001). The MDA levels in cortical regions of PTZ group were significantly higher than those of control animals (P < 0.001) (Figure 3). As shown in figure 3, pretreatment with both 100 and 1000 mg/kg of the extract resulted in a significant reduction in the free radical-mediated lipid peroxidation as indicated by a decrease in the MDA levels (P < 0.001 and P < 0.010, respectively). 

**Figure 4:**
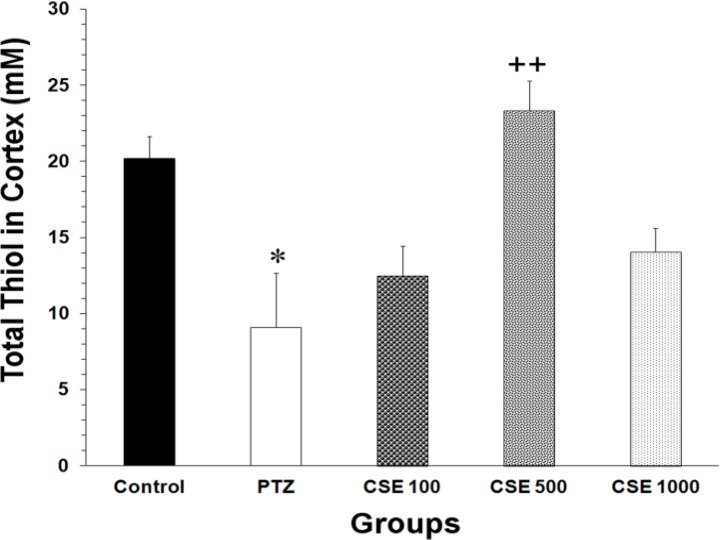
Comparison of the total SH groups in cortical tissues of control, pentylenetetrazole (PTZ), C. sativum extract (CSE) 100 mg/kg (CSE 100)-PTZ, CSE 500 mg/kg (CSE 500)-PTZ, CSE 1000 mg/kg (CSE 1000)-PTZ groups. The animals were treated with saline or CSE (100, 500 or 1000 mg/kg) before a single injection (90 mg/kg) of PTZ. The animals in the control group received saline instead of PTZ; ^*^ P < 0.0500 as compared to Control group;^ ++^ P < 0.0100 as compared to PTZ group

Data analysis using one-way ANOVA also showed that there was a significant difference between the groups in total thiol contents of cortical tissues (F_4,35_ = 6.78, P < 0.001). Following PTZ administration, a significant reduction in total SH groups in cortical samples was observed (P < 0.050, Figure 4). Pretreatment with 500 mg/kg of the extract prevented of decreased total thiol concentration in cortical tissues, as compared with PTZ group (P < 0.010).

**Figure 5:**
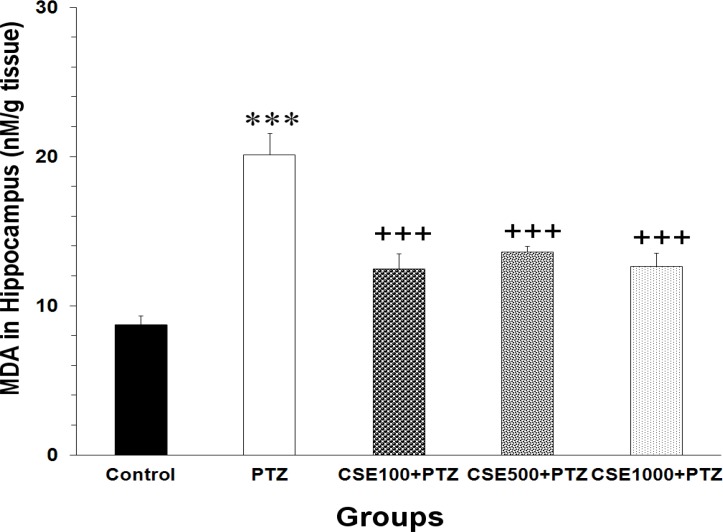
Comparison of the malondialdehyde (MDA) levels in hippocampal tissues of control, pentylenetetrazole (PTZ), C. sativum extract (CSE) 100 mg/kg (CSE 100)-PTZ, CSE 500 mg/kg (CSE 500)-PTZ, CSE 1000 mg/kg (CSE 1000)-PTZ groups. The animals were treated with saline or CSE (100, 500 or 1000 mg/kg) before a single injection (90 mg/kg) of PTZ. The animals in the control group received saline instead of PTZ; ^***^ P < 0.0010 as compared to control group; ^+++^ P < 0.001 as compared to PTZ group

Data analysis using one-way ANOVA showed that there was a significant difference between the groups in MDA concentrations of hippocampal tissues (F_4,35_ = 24.53, P < 0.0001). The MDA levels in the hippocampal regions of PTZ group were significantly higher than those of control animals (P < 0.001) (Figure 5). The results also showed that all three doses of CSE prevented the elevation of MDA concentration in hippocampal tissues (P < 0.001 for all, Figure 5).

**Figure 6:**
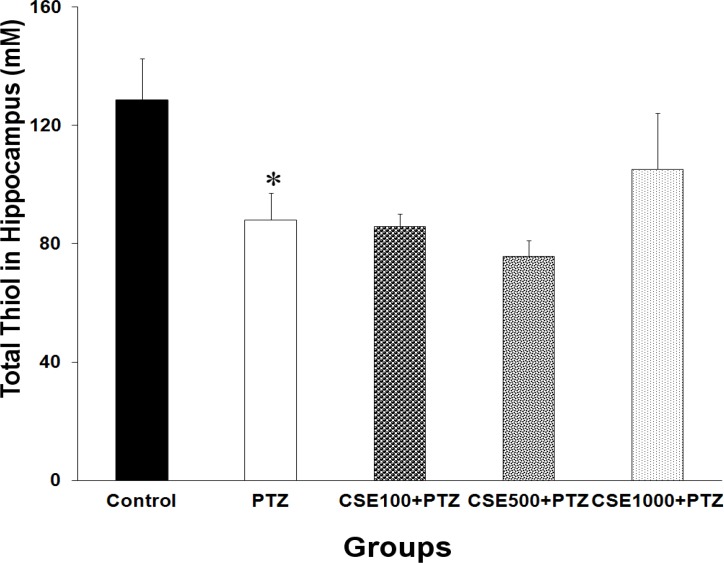
Comparison of the total SH groups in hippocampal tissues of control, pentylenetetrazole (PTZ), C. sativum extract (CSE) 100 mg/kg (CSE 100)-PTZ, CSE 500 mg/kg (CSE 500)-PTZ, CSE 1000 mg/kg (CSE 1000)-PTZ groups. The animals were treated with saline or CSE (100, 500 or 1000 mg/kg) before a single injection (90 mg/kg) of PTZ; The animals in control group received saline instead of PTZ; ^*^ P < 0.050 as compared to control group.

Data analysis using one-way ANOVA also showed that there was a significant difference between the groups in total thiol contents of hippocampal tissues (F_4,35_ = 3.19, P < 0.050). Following PTZ administration, a significant reduction in total SH groups in the hippocampal samples was observed (P < 0.050, Figure 6). There were no significant differences between CSE treated rats and PTZ group when total thiol content in hippocampal tissues was compared (Figure 6).

## Discussion

Oxidative stress is a basis etiology for many neurological and neurodegenerative disorders. Previous studies demonstrated that oxidative stress plays an important role in the pathogenesis of epileptic seizures.^3^^,^^4^^,^^31^ Elevation of free radical levels during seizures^7^^,^^31^ has been well documented therefore, it is suggested that oxidative stress has an important role in the brain damages due to epilepsy.^9^^,^^32^ Furthermore, the brain tissues oxidative damage contributes to the complications of seizures and epilepsy including cognitive, learning and memory impairments.^2^^,^^10^ In the present study the possible protective effects of C. sativum aerial parts on PTZ-induced seizures and the brain tissues oxidative damages was investigated. PTZ, is a selective inhibitor of the chloride channel which is coupled to the gamma-aminobutyric acid receptor.^33^ It is a well-known chemoconvulsant which is frequently used for evaluation of antiepileptic drugs.^34^^,^^35^ A high dose of PTZ induces a continued seizure activity which progress from mild myoclonic jerks to face and forelimbs clonus without loss of righting reflex (which is known as MCS), to clonic seizures of limbs with loss of righting reflex which is followed by full tonic extension of both forelimbs and hindlimbs (GTCS).^36^ PTZ has been repeatedly used in 90-100 mg/ kg to induce MCS and GTCS seizures.^9^^,^^22^^-^^25^^,^^27^^,^^28^ We also previously showed that PTZ-induced seizures are accompanied with brain tissues oxidative damage.^9^ The contribution of ROS to the neurotoxic effects of PTZ has also been suggested.^37^^,^^38^ Similarly, in the present study, we observed an increase in MDA levels and a reduction in total SH groups in the brains of the animals subjected to PTZ-induced seizure. Increase in ROS production, including superoxide anions, hydroxyl radicals, and hydrogen peroxide, in the brains subjected to seizures, have been well documented.^39^^,^^40^ It has been suggested that oxidative damages of brain tissues by free radicals may lead to psychiatric or cognitive problems such as depression, anxiety and memory loss.^31^^,^^41^ The decreasing in the life span, which has been reported in the epileptic persons may also be at least in part, due to oxidative damages.^42^ Oxidative stress has also been suggested as a link between aging and seizure.^43^ In the present study, we assessed the effect of the extract by studying its effect on lipid peroxidation, which was measured in terms of MDA concentrations. Studies with animal models using the MDA assay have generally reported an increased lipid peroxidation in the brain tissues in seizures and epilepsy.^37^^,^^44^ In our experiments, we observed a significant increase of lipid peroxidation in the both hippocampal and cortical tissues which was prevented by 100 and 1000 mg/kg of the extract however, 500 mg/kg of the extract prevented MDA elevation in hippocampal but not cortical tissues. These results are in consistent with the recently reported protective effects of the plant against hippocampal tissues oxidative damage.^45^^-^^47^ It has been previously suggested the brain regions are differently vulnerable to increased lipid peroxidation. For example Shila et al. showed that lipid peroxidation was increased to the highest level in hippocampus among the brain regions, followed by cortex in arsenic intoxicated rats which was attributed to the different levels of iron contents.^48^ Regarding the results of the present study, it seems that the plant extract was more effective to prevent of increased lipid peroxidation due to PTZ-induced seizures in hippocampal tissues compared to cortical tissues. In consistent with these results, Velaga et al. also reported that the hydroalcoholic extract of C. sativum seeds was more effective to decrease lipid peroxidation in hippocampus of lead intoxicated rats, compared to the cortical, cerebellum and brain stem tissues.^45^ It has also been reported that training exercise and vitamin E were more effective to reduce lipid peroxidation in the hippocampal tissues of aged rats compared with the cortical tissues.^49^ SH groups are also known to be sensitive to oxidative damage and depleted following an oxidative insult^50^ therefore, we studied the effect of the extract on total thiol concentrations in the brain tissues after seizures. Similar to other studies, thiol groups were decreased in the brain following seizures injury. As mentioned earlier, CSE prevented PTZ-induced thiol depletion only in the cortex and not hippocampus. One possible explanation for this observation might be due to brain regional-dependent glutathione (GSH) metabolism, as a major source of thiol group.^51^ It has been observed that brain GSH concentrations varied in the range of 0.2-10 mM.^52^ It was reported that GSH levels are highest in cortex, followed by hippocampus and brain stem.^53^^,^^54^ Consistent with this finding, some studies reported a strong antioxidant activity for C. sativum.^15^^-^^20^ Furthermore, we showed that this activity of C. sativum was accompanied by an anticonvulsant effect as it increased both MCS and GTCS latencies. In keeping with these observations, the anticonvulsant activity of several agents with antioxidant effect such as melatonin, vineatrol, trans-resveratrol and alpha lipoic acid have also been shown.^7^^,^^8^ Using pentobarbital-induced hypnosis animal model, we previously found that the injection of 50, 100, 200 and 400 mg/kg of hydroalcoholic extract of C. sativum before pentobarbital increased the sleep time of the rats.^12^ Even a higher dose of the extract has also been previously used in the rats without observation of any toxic effect.^55^ In the current study, therefore we used comparable doses of CSE 30 min before PTZ injection to test its anticonvulsive effect. Previously, Hosseinzadeh and Madanifard reported an anticonvulsant effect for the aqueous and ethanolic extracts of C. sativum seeds.^56^ The same effect was found by Emamghoreishi and Heidari-Hamedani for aqueous and hydroalcoholic extracts and essential oil of the seeds.^57^ Regarding the results of present study the beneficial effects of aerial parts of the plant on seizures and its complications such as brain tissues oxidative is suggested however, further studies using other animal models are needed to be done in the future. The electrophysiological studies are also suggested for future.

 However, no pharmacological studies have been yet evaluated the anticonvulsant activity of aerial parts of this plant. These parts of C. sativum are widely consumed as a vegetable all over the world. With the present study, we showed that aerial parts of C. sativum are effective in protection against seizures and oxidative stress induced by PTZ.

In the present study, the chemical compound(s) responsible for these effects of C. sativum were not identified and needs to be more investigated in future studies. The presence of the flavonoids such as quercitin has been reported in CSE.^58^ On the other hand, it has been shown that the flavonoids have considerable anticonvulsant effects.^59^^,^^60^ Regarding sedative and CNS depressant effects of falvonoids such as quercetin, these effects could be attributed to the affinity of the compounds for the central benzodiazepine receptors.^61^^-^^65^ The beneficial effect of linalool in PTZ as well glutamate-related seizure models has been suggested.^66^^,^^67^ It might be suggested the beneficial effects of the extract which was observed in the present study, might be at least in part, due to linalool which is one the main constituents of coriander and has considerable antioxidant effects.^68^

## Conclusion

The present data demonstrate that the hydroalcoholic extract of C. sativum aerial parts possesses anticonvulsant activity. This activity is accompanied by an antioxidant effect in brain tissues. Isolation of the active compound(s) from the extract needs to be done in the future.

## Notes:

**How to cite this article: **Karami R, Hosseini M, Mohammadpour T, Ghorbani A, Sadeghnia HR, Rakhshandeh H, et al. Effects of hydroalcoholic extract of Coriandrum sativum on oxidative damage in pentylenetetrazole-induced seizures in rats. Iran J Neurol 2015; 14(2): 59-66.
